# Bibliography

**Published:** 1916-06

**Authors:** 


### BIBLIOGRAPHY

INTERNAL ANATOMY OF THE FACE. The second edition of Cryer's Internal Anatomy of the Face has just been published by Lea and Febiger. It is greatly enlarged and much improved in typography and many new and excellent illustrations. Like its predecessor it is a most useful addition to dental literature. It is unique in books on scientific subjects, as it illustrates and describes the variant rather than the typal. The author's idea is that the typal forms are so rarely met with in practical surgery and the variations are so common that for the practitioner he should familiarize himself with the atypical. Many new illustrations of these conditions are added in this edition which increase the value of this work to the practitioner. There are included a number of excellent X-ray illustrations which supplement the well known section specimens of the former edition, and carry the ideas of author a step farther. The author and publishers have certainly placed the profession under great obligations for so excellent a help in all lines of dental surgery and therapeutics. We can hardly see how any up-to-date dentist can afford to be without this book, as it meets in a perfectly satisfactory manner many of the needs of present-day practice.

